# Initiation of Psychotropic Medication after Partner Bereavement: A Matched Cohort Study

**DOI:** 10.1371/journal.pone.0077734

**Published:** 2013-11-05

**Authors:** Sunil M. Shah, Iain M. Carey, Tess Harris, Stephen DeWilde, Christina R. Victor, Derek G. Cook

**Affiliations:** 1 Division of Population Health Sciences and Education, St George’s University of London, London, United Kingdom; 2 School of Health Sciences and Social Care, Brunel University, Uxbridge, Middlesex, United Kindom; University of Pennsylvania, United States of America

## Abstract

**Background:**

Recent changes to diagnostic criteria for depression in DSM-5 remove the bereavement exclusion, allowing earlier diagnosis following bereavement. Evaluation of the potential effect of this change requires an understanding of existing psychotropic medication prescribing by non-specialists after bereavement.

**Aims:**

To describe initiation of psychotropic medication in the first year after partner bereavement.

**Methods:**

In a UK primary care database, we identified 21,122 individuals aged 60 and over with partner bereavement and no psychotropic drug use in the previous year. Prescribing (anxiolytic/hypnotic, antidepressant, antipsychotic) after bereavement was compared to age, sex and practice matched controls.

**Results:**

The risks of receiving a new psychotropic prescription within two and twelve months of bereavement were 9.5% (95% CI 9.1 to 9.9%) and 17.9% (17.3 to 18.4%) respectively; an excess risk of initiation in the first year of 12.4% compared to non-bereaved controls. Anxiolytic/hypnotic and antidepressant initiation rates were highest in the first two months. In this period, the hazard ratio for initiation of anxiolytics/hypnotics was 16.7 (95% CI 14.7 to 18.9) and for antidepressants was 5.6 (4.7 to 6.7) compared to non-bereaved controls. 13.3% of those started on anxiolytics/hypnotics within two months continued to receive this drug class at one year. The marked variation in background family practice prescribing of anxiolytics/hypnotics was the strongest determinant of their initiation in the first two months after bereavement.

**Conclusion:**

Almost one in five older people received a new psychotropic drug prescription in the year after bereavement. The early increase and trend in antidepressant use after bereavement suggests some clinicians did not adhere to the bereavement exclusion, with implications for its recent removal in DSM-5. Family practice variation in use of anxiolytics/hypnotics suggests uncertainty over their role in bereavement with the potential for inappropriate long term use.

## Introduction

Bereavement and grief are near universal human experiences which adversely impact on physical and psychological health [1]. Recent changes in diagnostic criteria for depression after bereavement have highlighted a polarised debate over the role of medical care in managing the psychological impact of bereavement [2]. Existing criteria, also implemented in the UK, limited a diagnosis of a major depressive disorder within two months of bereavement to those with marked symptoms uncharacteristic of normal grief [3], [4]. This bereavement exclusion was intended to prevent over diagnosis of depression which may be difficult to distinguish from normal grief; a self-limiting process for the majority [3], [4]. The change to the Diagnostic and Statistical Manual of Mental Disorders **(**DSM**)** allows a diagnosis of depression at any time after bereavement, based on standard depression criteria, but maintains the position that acute grief is not a mental disorder [4].

Although current discussion has focussed on depression, the appropriateness of anxiolytic/hypnotic prescribing after bereavement has also been questioned, with concern over long-term use and blunting of normal grief responses [5], [6]. Despite this, there is limited information on current prescribing practice for unselected bereaved individuals [7], [8]. Specifically, it is not known whether the bereavement exclusion was followed for antidepressant prescribing and whether hypnotic use is appropriately time-limited. This information is important as the current debate over changes to DSM-5 is based on detailed assessment of psychological symptoms after bereavement and specialist assessment, whilst the majority of prescribing after bereavement in many countries, such as the UK, is undertaken in primary care. It is likely that the circumstances and specificity of diagnostic decisions will be different in primary care, which will mean that the consequences of changes in depression criteria may not be predictable. Recent studies have reported increased use of psychotropic medication after bereavement but have not directly examined the pattern of initiation of specific drug classes in relation to current guidance [7], [8]. This is a key gap in the evidence base to help understand the practical implications of changes in diagnostic criteria.

In this study, we describe initiation, continuation and determinants of psychotropic medication prescribing in the first year after partner bereavement in a large primary care based sample of older people in the United Kingdom. Specifically, we examine whether antidepressant prescribing was limited by primary care physicians in the first two months after bereavement, as an indicator of adherence to the existing bereavement exclusion, and whether prescribing of anxiolytics/hypnotics after bereavement leads to long-term use.

## Methods

### Data Source

The Health Improvement Network (THIN) is an established primary care database which collects anonymised data from UK general practices and includes a full record of diagnoses and prescribing [9], [10]. The THIN database includes approximately 6% of the UK population. A feature of the THIN database is the family number which allows practices to identify patients who live in the same household [11].

### Ethics Statement

The National Health Service South East Multicentre Research Ethical Committee approved the THIN scheme to collect anonymous patient data in 2002. This approval allows collection of anonymised patient data for research use without individual patient consent but allows patients to opt out of the scheme. This specific study was further approved by the South-East NHS Research Ethics Committee (11/H1102/3).

### Subjects

Out of 495 practices providing data to the THIN scheme at study inception in 2010, we included all 401 practices active in THIN scheme between 2005 and 2008 which were able to provide data for at least one year. We used a historical patient file to identify the household composition for a cohort of older patients aged 60 and over on a specified (index) date between 2005 and 2008 for each practice (n = 672,543).

We developed an algorithm, described in detail elsewhere, to identify 171,720 couples with an age difference of ten years or less [12]. We have confirmed the validity of our algorithm by comparison with national representative surveys in England which confirm that 99.4% of couples selected using our criteria identify themselves as married or cohabiting [12].

### Bereavement

Couples were followed in the primary care record from the index date for their practice to their last practice data collection date up to September 2011. The timing of bereavement was identified through the record of death in the deceased partner’s primary care record. 31,370 older people experienced bereavement during follow up and our analysis is restricted to 29,548 aged between 60 and 89 at beginning of follow up.

### Psychotropic Drug Prescribing

We identified any prescription for anxiolytics, hypnotics, antidepressants and antipsychotics in the primary care record. Low dose amitriptyline (<50 mg) prescribing was examined separately and not included in our main antidepressant or composite psychotropic drug group. In the UK, low dose amitriptyline may be prescribed for pain management or as a hypnotic. We defined initiation as a new prescription of any of these drug classes in a bereaved individual who had not received any psychotropic medication in the year before bereavement.

### Study Design

Our main design was a matched cohort study which compared rates of prescribing in bereaved individuals to those in an age, sex, practice and period matched cohort. This allowed description of cumulative, relative, and absolute differences in initiation of prescribing in the first year after bereavement compared to non-bereaved individuals. Our main analysis is focused on bereaved individuals and matched controls who were not in receipt of any psychotropic drugs in the year before bereavement. This allows a description of the direct effect of bereavement on initiation of medication.

### Matched Control Groups

Two distinct control groups of non-bereaved individuals were identified who were registered on the bereavement date of their matched counterpart with matching on age (in five year age bands), sex and practice. The first group matched all bereaved individuals and was used for preliminary description of prescribing patterns before and after bereavement. The second group matched bereaved individuals who had not received psychotropic medication in the year before bereavement to controls who had also not received psychotropic medication. This second group was used for our main analysis of medication initiation after bereavement. Up to three controls were matched for every bereaved individual.

### Participants

Of the 29,548 bereaved individuals aged 60 to 89 at baseline, 21,668 (73%) had not received a prescription of any psychotropic medication in the year before bereavement. For our first matched groups, 28,800 (97.5%) of all bereaved individuals were successfully matched with at least one non-bereaved control giving 80,471 age, sex and practice matched non-bereaved controls For our second matched group, 21,122 (97.5%) of bereaved individuals free of psychotropic medication in the year before bereavement were successfully matched with at least one non-bereaved control who was also free of psychotropic medication a year before the bereavement date, giving 59,280 age, sex and practice matched non-bereaved controls. All analyses only include bereaved individuals with at least one matched control.

### Analysis

Initial graphical analysis of prescribing patterns, before and after bereavement, identified the proportion of patients still registered that had received a prescription in the last month (30 days). Descriptive analysis presents Kaplan-Meier estimates of the probability of bereaved individuals receiving a new first prescription of each class of drug in the two (60 days), six (180 days) and twelve (365 days) months after bereavement with individuals censored from the analysis on death or deregistration.

Subsequent analysis compared initiation rates in consecutive periods (0–2 months, 2–6 months and 6–12 months) in the first year after bereavement for bereaved individuals and the matched control group. Hazard ratios for initiation are based on a Cox proportional hazards survival model, stratified by matched sets.

### Continuation

Continuation of prescribing at the first anniversary of bereavement, among those initiated within two months (60 days), was defined as receipt of a prescription for the same drug class in the last three months (90 days) of the year. We excluded patients who had died or deregistered at one year. The mean number of prescriptions of each drug class in the first year after bereavement is also presented as an alternate measure of continuation. Guidelines on use of anxiolytics and hypnotics suggest that they should be time-limited for periods of two to four weeks while antidepressant therapy is recommended for at least six months after remission [13].

### Predictors of Initiation

Predictors of initiation of anxiolytics/hypnotics, within two months, and antidepressants within six months of bereavement were examined and compared to predictors in non-bereaved controls. The timeframe for anxiolytics/hypnotics was based on the existing DSM-IV threshold for the acute grief period (2 months) and for antidepressants based on the proposed threshold for diagnosis of complicated grief (after 6 months).

Our main measure of comorbidity was the Charlson Index, a validated score that weights 17 chronic physical conditions with a score of 1 to 6 [14]. The Townsend Index, a composite small area ecological measure of deprivation, was assigned to couples based on their postcode and summarised as quintiles based on national ranking [15]. Season of bereavement was based on standard UK definitions, with December to March defined as Winter [16]. Unexpected bereavements were defined as bereavements where the deceased partner had no recorded Charlson comorbidity or other disabling condition more than 30 days before their death [17].

General practice prescribing characteristics were based on background prescribing of anxiolytics/hypnotics or antidepressants to non-bereaved couples in 2008. Practices were classified in quintiles, from lowest to highest, based on the proportion of non-bereaved individuals who received a prescription of the drug class during the year.

Predictors of psychotropic drug initiation were examined using a Cox proportional hazards model with separate models for bereaved and non-bereaved individuals. Hazard ratios were adjusted for clustering at practice level using the sandwich estimator to produce robust standard errors. P values for an interaction between bereavement status and individual predictors were derived from a combined model including both bereaved individuals and non-bereaved controls.

## Results

### Follow Up

Mean age at bereavement was 76.2 years (range 60–95). Among the bereaved with no psychotropic medication use in the year before bereavement, 16,113/21,122 (76.3%) contributed a full year of post-bereavement follow up, 633 (3.0%) died within one year, 789 (3.7%) left their practice in the first year and 3,587 (17.0%) were bereaved less than a year before end of follow up for their practice. In the matched control group, 46,882/59,280 (79.1%) contributed a full year of follow up. Irrespective of follow up time, 3,547/21,122 bereaved individuals and 2,907/59,280 controls received a new prescription of a psychotropic medication in the first year after bereavement.

### Pattern of Psychotropic Drug Prescribing

**Figure 1:**
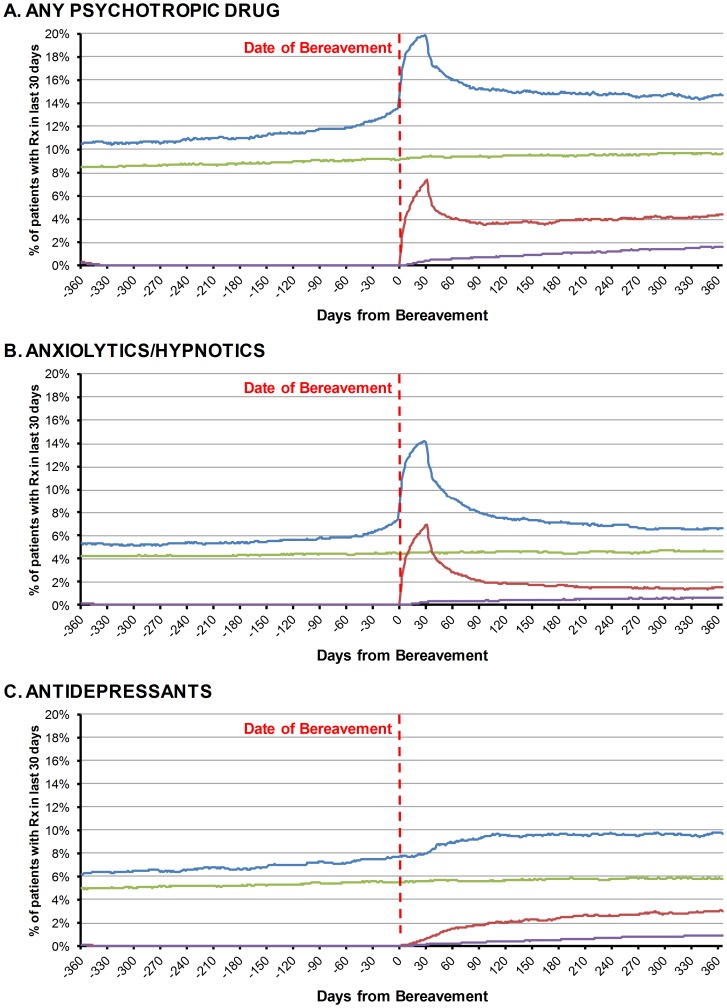
Proportion of Patients in Receipt of Psychotropic Medication in the Year Before and After Bereavement. Blue: All Bereaved (n = 29,548) Green: All Non-Bereaved (n = 80,471) Red: Bereaved without psychotropic medication in year before bereavement (n = 21,122) Purple: Non-Bereaved without psychotropic medication in year before bereavement (n = 59,280) Daily percentages are based on a denominator of patients still registered on that day.

Figure 1 shows the temporal pattern of medication use before and after bereavement in (i) all bereaved individuals and (ii) those without any prescription in the year before bereavement (initiation), with their respective matched control group. For all medication, bereaved individuals experienced higher prescribing before bereavement (figure 1a). After bereavement, there was a sharp rise in all prescribing in the first two months followed by decline but levels remained persistently elevated throughout the first year compared to both the control group and pre-bereavement prescribing. This overall pattern largely reflects anxiolytic/hypnotic prescribing (figure 1b). Antidepressant prescribing exhibited a slower rise in the first two months and a more persistent increase over the first year after bereavement (figure 1c).

### Initiation of Psychotropic Medication

**Figure 2:**
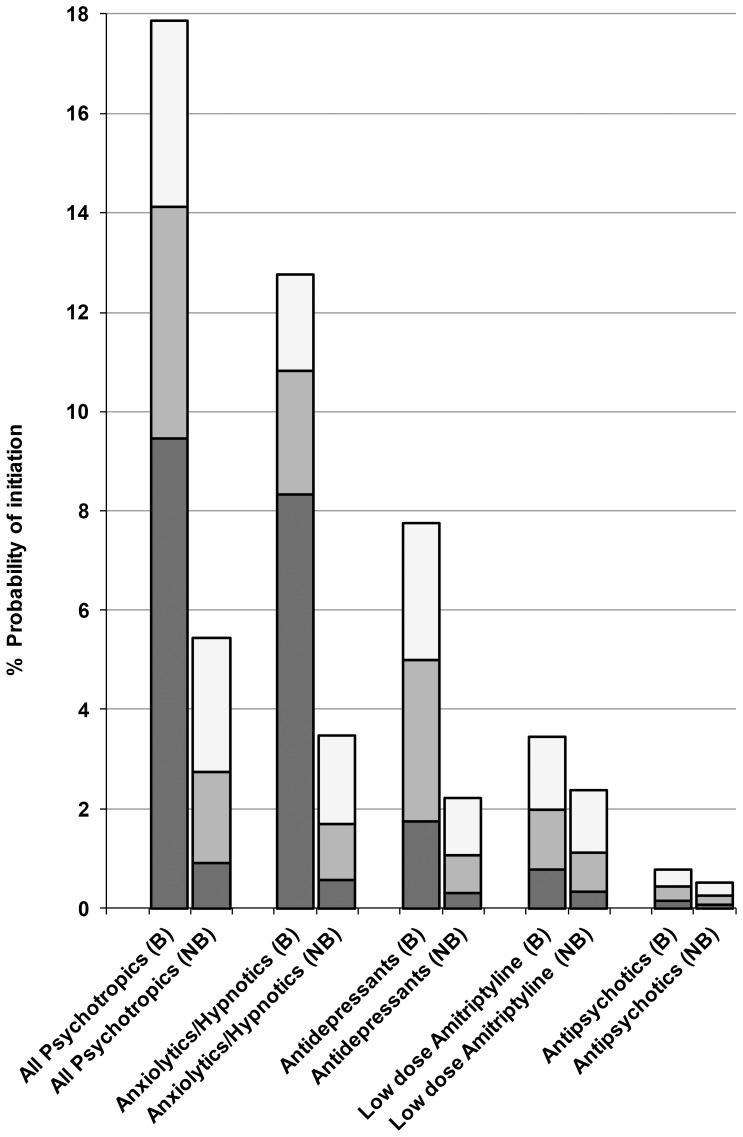
Probability of Initiation (%) of Psychotropic Drugs in the Year after Bereavement in Bereaved (B) and Non Bereaved (NB) Patients. Initiation probabilities during the first year after bereavement are derived from Kaplan Meier estimates and divided into the following periods Light Grey: 6–12 months Medium Grey: 2–6 months Dark Grey: 0–2 months.

The probability of initiation of medications during the first year after bereavement is shown in figure 2. 9.5% (95% CI 9.1 to 9.9%) of bereaved individuals were newly prescribed any psychotropic medication in the two months after bereavement and 17.9% (17.3 to 18.4%) within a year of bereavement. This compares to 0.9% (0.8 to 1.0%) and 5.4% (5.3 to 5.6%) for the non-bereaved controls. This is an absolute difference in the probability of initiation of 8.6% in the first two months and 12.4% at one year between the bereaved and non-bereaved groups.

Among the bereaved, the risks of receiving a new prescription of anxiolytics/hypnotics and antidepressants in the first two months were 8.3% (8.0 to 8.7%) and 1.8% (1.6 to 1.9%) respectively compared to 0.6% (0.5 to 0.6%) and 0.3% (0.3 to 0.4%) of non-bereaved controls (figure 2). In the two months after bereavement, the most common first prescribed medications were Zopiclone (31%), Diazepam (26%) and Temazepam (22%).

### Matched Comparison

Further analysis confirmed the elevated initiation rates throughout the first year for all classes of psychotropic drugs (table 1). In matched analysis, compared to the non-bereaved controls, the increased absolute and relative risk of receiving a prescription was most marked in the first two months for anxiolytics/hypnotics (absolute rate difference (ARD) 3.92% per month, HR 16·7) and antidepressants (ARD 0.7% per month, HR 5·6). This effect persisted for antidepressants between two and six months (ARD 0.64% per month, HR 4·3) but attenuated between six and twelve months (ARD 0.29% per month, HR 2·5). The trend in both absolute and relative excess rate of antidepressant prescribing showed the greatest increase in the first two months of prescribing, the period of the bereavement exclusion. Analysis restricted to patients with a full year of follow up gave the same findings.

**Table 1:** Psychotropic Medication Initiation in the First Year after Bereavement Compared to Non-Bereaved Patients.

DrugGroup	Time afterbereavement	Bereaved(n = 21,122)		Non-Bereaved(n = 59,280)		Bereaved (n = 21,122) vs. Non-Bereaved (n = 59,280)			
		Numberinitiating	InitiationRate/100PMAR^a^	Numberinitiating	InitiationRate/100PMAR^a^	Absolutedifference/100PMAR^a^	95% CI	HazardRatio	95% CI
Anxiolytics/Hypnotics	0–2 months	1744	4.20	329	0.28	+3.92	3.72 to 4.12	16.68	14.73 to 18.88
	2–6 months	488	0.68	634	0.29	+0.40	0.33 to 0.46	2.45	2.16 to 2.76
	6–12 months	336	0.35	887	0.29	+0.06	0.02 to 0.10	1.21	1.06 to 1.38
	0–12 months	2,568	1.12	1,850	0.28	+0.84	0.79 to 0.88	4.50	4.22 to 4.79
Antidepressants^b^	0–2 months	361	0.87	181	0.15	+0.72	0.62 to 0.81	5.58	4.65 to 6.70
	2–6 months	635	0.83	422	0.19	+0.64	0.57 to 0.70	4.29	3.78 to 4.87
	6–12 months	489	0.48	585	0.19	+0.29	0.24 to 0.33	2.53	2.23 to 2.87
	0–12 months	1,485	0.65	1,188	0.18	+0.47	0.43 to 0.50	3.66	3.38 to 3.96
Low Dose Amitriptyline only	0–2 months	162	0.39	200	0.17	+0.22	0.15 to 0.28	2.36	1.92 to 2.92
	2–6 months	237	0.31	437	0.20	+0.11	0.07 to 0.15	1.59	1.35 to 1.86
	6–12 months	253	0.24	624	0.20	+0.04	0.00 to 0.07	1.15	0.99 to 1.33
	0–12 months	652	0.29	1,261	0.19	+0.09	0.07 to 0.12	1.49	1.36 to 1.65
Antipsychotics	0–2 months	33	0.08	39	0.03	+0.05	0.02 to 0.08	2.09	1.30 to 3.36
	2–6 months	53	0.07	104	0.05	+0.02	0.00 to 0.04	1.42	1.01 to 1.99
	6–12 months	59	0.05	133	0.04	+0.01	−0.01 to 0.03	1.23	0.90 to 1.68
	0–12 months	145	0.06	276	0.04	+0.02	0.00 to 0.03	1.43	1.16 to 1.76
All Psychotropics^b^	0–2 months	1978	4.76	526	0.45	+4.31	4.10 to 4.52	11.82	10.68 to 13.08
	2–6 months	915	1.29	1024	0.46	+0.83	0.74 to 0.92	2.77	2.52 to 3.04
	6–12 months	654	0.71	1357	0.45	+0.26	0.20 to 0.32	1.58	1.43 to 1.74
	0–12 months	3,547	1.54	2,907	0.45	+1.10	1.04 to 1.15	3.96	3.76 to 4.17

a Person months at risk (% Initiation per month).

b Excludes low dose amitriptyline.

The pattern of low dose amitriptyline use after bereavement was different from other antidepressants with a less marked rise. Similarly, antipsychotic prescribing showed a very small absolute increase after bereavement. Further adjustment of hazard ratios for deprivation and comorbidity made no difference to our findings for any prescribing outcome.

### Continuation

Overall, 25.2% (395/1566) who started a psychotropic medication within two months were receiving some psychotropic medication at one year (table 2). 13.3% (185/1392) continued to receive anxiolytics/hypnotics, with an average of 2.55 prescriptions per patient, and 43.9% (123/280) continued to receive antidepressants. Continuation was similar in bereaved and non-bereaved individuals.

**Table 2:** One Year Continuation of Psychotropic Medication Initiated in the First Two Months after Bereavement.

	Bereaved (n = 21,122)					Non-Bereaved (n = 59,280)				
	Initiated within 2 months	Active at 1 year*	Rx At One Year†	% of active	Mean Prescriptions (SE)	Initiated within 2 months	Active at 1 year*	Rx At One Year†	% of active	Mean Prescriptions (SE)
Anxiolytics/Hypnotics	1,744	1,392	185	13.3%	2.55 (0.08)	329	236	34	14.4%	2.18 (0.17)
Antidepressants	361	280	123	43.9%	6.02 (0.36)	181	129	54	41.9%	6.41 (0.67)
All	1,978	1,566	395	25.2%	3.81 (0.12)	526	362	108	29.8%	4.31 (0.32)

* Alive and registered with practice on first anniversary of bereavement date.

† Received same class of medication within 90 days of anniversary of bereavement.

### Predictors of Initiation

Predictors of initiation of anxiolytics/hypnotics in the first two months are shown for bereaved individuals in table 3. Anxiolytic/hypnotic prescribing was slightly more common in women and lower in older individuals but deprivation or comorbidity had little influence on prescribing. This contrasts with prescribing in the non-bereaved where comorbidity was a strong predictor and prescribing increased with age. Practice background prescribing was the strongest independent predictor of initiation with a hazard ratio of 1.70 (95% CI 1.43 to 2.03) for bereaved individuals in the highest quintile practices compared to those in the lowest prescribing quintile. Absolute Initiation after bereavement was markedly higher in practices with high background prescribing; 11.2% in practices in the highest quintile compared to 6.2% in those in the lowest quintile.

**Table 3:** Adjusted Hazard Ratios for Predictors of Initiation of Anxiolytics/Hypnotics within 2 months of Bereavement.

Group	Bereaved (n = 21,122)					Non-Bereaved* (n = 59,280)	
	Total	Initia-tors	%	HR^a^	95% CI	HR^a^	Interaction Test^b^
Gender							P = 0.75
Men	8,186	596	7.3%	**1**	_	**1**	
Women	12,936	1,148	8.9%	**1**.**18**	1.07 to 1.31	**1**.**41**	
Age at bereavement							P<0.001
60–64	1,279	133	10.4%	**1**.**24**	1.01 to 1.50	**1.08**	
65–69	3,269	325	9.9%	**1.15**	1.00 to 1.34	**0.87**	
70–74	4,390	372	8.5%	**0.99**	0.87 to 1.14	**0.68**	
75–79	5,004	426	8.5%	**1**	_	**1**	
80–84	4,410	327	7.4%	**0**.**89**	0.77 to 1.03	**0.93**	
85+	2,770	161	5.8%	**0**.**70**	0.59 to 0.84	**1.24**	
Townsend Quintile (Deprivation)							P = 0.98
1 (Least Deprived)	5,859	474	8.1%	**1**	_	**1**	
2	5,338	436	8.2%	**0**.**99**	0.87 to 1.13	**1**.**10**	
3	4,316	331	7.7%	**0**.**92**	0.80 to 1.06	**1**.**02**	
4	3,406	300	8.8%	**1**.**05**	0.91 to 1.22	**1**.**19**	
5 (Most Deprived)	1,777	167	9.4%	**1**.**09**	0.91 to 1.30	**1**.**27**	
Missing	426	36	8.5%	**0**.**98**	0.70 to 1.38	**0**.**85**	
Charlson Index (Comorbidity)							P<0.001
0	10,279	844	8.2%	**1**	_	**1**	
1	5,114	443	8.7%	**1**.**09**	0.97 to 1.22	**1**.**26**	
2–3	4,461	379	8.2%	**1**.**07**	0.94 to 1.21	**1**.**27**	
4+	1,088	78	7.2%	**0**.**96**	0.76 to 1.22	**2**.**59**	
Depression							P = 0.77
No History	19,559	1,545	7.9%	**1**	_	**1**	
History	1,563	199	12.7%	**1**.**60**	1.38 to 1.86	**1**.**52**	
Bereavement Expectation							n/a
Unexpected	2,805	251	9.0%	**1**.**15**	1.01 to 1.32	**_**	
Known Partner Comorbidity	18,317	1,493	8.2%	**1**	_	**_**	
Bereavement Season							P = 0.38
Non-Winter	13,676	1,094	8.0%	**1**	_	**1**	
Winter	7,446	650	8.7%	**1**.**11**	1.01 to 1.22	**0**.**99**	
Practice Anx/Hyp Prescribing^C^							P = 0.80
I (0 to 5.86%)	4,009	248	6.2%	**1**	_	**1**	
II (5.86 to 7.09%)	4,595	365	7.9%	**1**.**29**	1.09 to 1.52	**1**.**10**	
III (7.09 to 8.31%)	4,327	324	7.5%	**1**.**22**	1.03 to 1.45	**1**.**10**	
IV (8.31 to 10.49%)	4,885	436	8.9%	**1**.**49**	1.27 to 1.75	**1**.**35**	
V (10.49 and above)	3,306	371	11.2%	**1**.**70**	1.43 to 2.03	**1**.**89**	

a Hazard ratios adjusted for all determinants in table, UK Region and Year.

b Comparing Association in Bereaved and Non-Bereaved Groups.

c Quintiles of annual practice prescribing to non-bereaved couples (% of patients in receipt of this drug class).

* Full data on non-bereaved analysis is presented in table S1.

For initiation of antidepressants in the first six months (table 4), female gender, comorbidity, deprivation and a history of depression predicted prescribing but age had no clear trend. In the non-bereaved, determinants of prescribing were similar except that increasing age increased the likelihood of prescribing and deprivation was a stronger predictor. Background practice prescribing of antidepressants strongly influenced antidepressant prescribing after bereavement. For both classes of drugs, unexpected bereavement did not strongly influence the likelihood of receiving medication.

**Table 4:** Adjusted Hazard Ratios for Predictors of Initiation of Antidepressants within 6 months of Bereavement.

Group	Bereaved (n = 21,122)					Non-Bereaved* (n = 59,280)	
	Total	Initia-tors	%	HR^a^	95% CI	HR^a^	Interaction Test^b^
Gender							P = 0.13
Men	8,186	336	4.1%	**1**	_	**1**	
Women	12,936	660	5.1%	**1.30**	1.13 to 1.48	**1**.**62**	
Age at bereavement							P<0.001
60–64	1,279	79	6.2%	**1.30**	1.01 to 1.69	**0.73**	
65–69	3,269	155	4.7%	**0.98**	0.80 to 1.20	**0.77**	
70–74	4,390	181	4.1%	**0.85**	0.70 to 1.03	**0.77**	
75–79	5,004	243	4.9%	**1**	_	**1**	
80–84	4,410	192	4.4%	**0.91**	0.75 to 1.10	**1.24**	
85+	2,770	146	5.3%	**1.14**	0.93 to 1.40	**1.59**	
Townsend Quintile (Deprivation)							P = 0.003
1 (Least Deprived)	5,859	242	4.1%	**1**	_	**1**	
2	5,338	253	4.7%	**1**.**15**	0.97 to 1.38	**1**.**32**	
3	4,316	183	4.2%	**1**.**03**	0.85 to 1.24	**1**.**50**	
4	3,406	196	5.8%	**1**.**38**	1.14 to 1.68	**1**.**03**	
5 (Most Deprived)	1,777	107	6.0%	**1**.**39**	1.10 to 1.76	**1**.**86**	
Missing	426	15	3.5%	**0**.**86**	0.51 to 1.45	**0**.**74**	
Charlson Index (Comorbidity)							P = 0.50
0	10,279	426	4.1%	**1**	_	**1**	
1	5,114	238	4.7%	**1**.**13**	0.96 to 1.33	**1**.**22**	
2–3	4,641	255	5.5%	**1**.**38**	1.18 to 1.62	**1**.**46**	
4+	1,088	77	7.1%	**1**.**85**	1.45 to 2.37	**2**.**04**	
Depression							P = 0.37
No History	19,559	870	4.5%	**1**	_	**1**	
History	1,563	126	8.1%	**1**.**75**	1.45 to 2.11	**2**.**04**	
Bereavement Expectation							n/a
Unexpected	2,805	137	4.9%	**1**.**10**	0.92 to 1.32	**_**	
Known Partner Comorbidity	18,317	859	4.7%	**1**	_	**_**	
Bereavement Season							P = 0.80
Non-Winter	13,676	627	4.6%	**1**	_	**1**	
Winter	7,446	369	5.0%	**1**.**03**	0.90 to 1.17	**1**.**02**	
Practice Antidepresant Prescribing^c^							P = 0.58
I (0 to 6.56%)	3,647	132	3.6%	**1**	_	**1**	
II (6.56 to 7.88%)	5,192	237	4.6%	**1**.**29**	1.04 to 1.60	**1**.**12**	
III (7.88 to 9.00%)	4,684	224	4.8%	**1**.**35**	1.09 to 1.68	**1**.**49**	
IV (9.00 to 10.66%)	4,429	224	5.1%	**1**.**41**	1.13 to 1.76	**1**.**41**	
V (10.66 and above)	3,170	179	5.7%	**1.60**	1.25 to 2.04	**1**.**52**	

a Hazard ratios adjusted for all determinants in table, UK Region and Year.

b Comparing Association in Bereaved and Non-Bereaved Groups.

c Quintiles of annual practice prescribing to non-bereaved couples (% of patients in receipt of this drug class).

* Full data on non-bereaved analysis is presented in table S1.

## Discussion

We have identified a marked increase in initiation of both anxiolytics and antidepressants after bereavement with almost one in five older people receiving a new psychotropic medication in the first year after death of their partner. For antidepressants, comparison of prescribing in the first two months after bereavement to subsequent months, suggests that clinicians do not adhere to the bereavement exclusion. For both anxiolytics/hypnotics and antidepressants, the likelihood of prescribing after bereavement is strongly determined by practice prescribing propensity and, for anxiolytics/hypnotics, subsequent long-term use is not uncommon.

### Strengths and Weaknesses

Our study describes prescribing in a large unselected community based population across the United Kingdom. Our matched analysis allows direct comparison between bereaved and non-bereaved individuals taking account of practice variation and temporal trends.

We restricted our main analysis to individuals who were free of any psychotropic prescribing in the year before bereavement to exclude individuals with pre-bereavement psychological problems which may worsen after bereavement. This means that our estimates of initiation after bereavement are conservative as individuals receiving one class of psychotropic drug were more likely to start another type after bereavement.

For depression, we have chosen to report on prescribing outcomes rather than recorded depression diagnoses. We believe that this approach is preferable as it avoids concerns over variations in diagnostic recording and coding between clinicians, especially if these are related to knowledge of a recent bereavement. We cannot exclude the possibility that patients receiving antidepressants in the first two months after bereavement met the stringent diagnostic criteria with marked functional impairment or severe symptoms required in DSM-IV but this is unlikely. Crucially, the fall in antidepressant prescribing in the next four month period is inconsistent with this explanation.

Our analysis does not take account of receipt of psychological therapy either before or after bereavement. Uptake of this intervention is not as consistently recorded as prescribing and the main focus of our paper was psychotropic drug use. It is possible that differential use of psychological therapies could explain some of the differences between bereaved and non-bereaved individuals but they are unlikely to explain the large differences observed. Similarly, our analysis cannot distinguish patient or doctor initiated prescribing. It is plausible that prescribing of anxiolytic medication may be requested by bereaved individuals or their relatives and this needs to be considered in interpreting the policy and clinical implications of our findings.

Our results will be influenced by loss to follow up due to death and deregistration. This is partially addressed by our use of censored analysis and comparison with a matched control group but this cannot account for informative loss to follow up. However, for these loses to introduce bias would require a complex interaction between bereavement, the likelihood of receiving psychotropic medication and loss to follow up which we feel is unlikely.

### Context

We are only aware of one recent large study which has examined psychotropic prescribing in unselected bereaved individuals. This Swedish study found modestly increased risks of receiving any psychotropic medication in the year after bereavement but did not further describe this effect by drug type or time since bereavement [7]. A Danish study examined prescribing after myocardial infarction and reported on monthly antidepressant and benzodiazepine prescribing after bereavement with a similar pattern to our study [8].

### Implications

#### Anxiolytic and Hypnotics

Our study confirms that prescribing of anxiolytics/hypnotics is common after bereavement. In absolute terms, there is a 9.3% higher risk of receiving these drugs in the year after bereavement (derived from figure 2). Their short-term use is not contra-indicated and pharmacological relief of distress or insomnia may be viewed as a compassionate clinical response [5], [13]. However, there is a concern that use of these drugs may inhibit normal grieving. A small RCT, with benzodiazepines, did not find that their use altered the psychological response to bereavement but reported prolonged sleep disturbance [5]. Evidence from studies on post-traumatic stress disorder also raise concerns over the long terms effects of benzodiazepines used to manage initial distress after traumatic events [18].

Our findings raise other concerns. The high continuation rate of these drugs is counter to current guidelines, which only support short-term use to minimise the well-established harm from long-term use [19], [20]. Initiation after bereavement may be an important gateway to long-term use and dependency [6]. In our analysis, at least one in eight patients continued medication at one year with a risk of continuation similar to prescribing outside the bereavement period. This risk needs to be considered in determining the benefit to harm ratio for prescribing after a common event during the life course of older people.

A further concern from our study is the large practice variation, which is not explained by differences in deprivation or patient comorbidity, and apparently determined by clinician prescribing propensity. Such variation suggests that prescribing indications are far from consistent and that prescribing habit, rather than individual circumstances, determine whether an individual receives medication after bereavement.

#### Antidepressants

In absolute terms, the effect of bereavement on antidepressant prescribing is relatively modest, with a 1.4% increased risk in the first two months and 5.5% in the first year (derived from figure 2), but our findings gives an important insight into clinical practice after bereavement and current adherence to the bereavement exclusion. The risk of antidepressant prescribing was greatest in the first two months after bereavement and decreased slightly by six months and markedly by one year which suggests that the previous bereavement exclusion had little effect on clinician decision making. A reluctance to diagnose, and pharmacologically treat, depression in the first two months after bereavement, limiting treatment to those with severe symptoms, would delay the increase in prescribing and see a marked increase after two months. This means, at least in the UK, removal of the bereavement exclusion from criteria for diagnosis of major depressive episodes may not be helpful and potentially promote excessive prescribing. The main driver for this change is a desire not to withhold effective treatment after bereavement [4]. As the previous bereavement exclusion was not measurably reducing or delaying prescribing, this recent change to the diagnostic criteria may lead to excessive treatment if diagnostic specificity is poorer in primary care, where it may be more difficult to distinguish between normal grief and depression.

It is important that any change to diagnostic criteria, which further encourage prescribing, are supported by an evidence base on the effectiveness and harm from treatment to allow informed decisions by patients and clinicians. The current evidence on antidepressants for early bereavement related depression is limited, but does suggest that treatment may relieve depression symptoms but not manifestations of grief [3], [21], [22]. This limited evidence base stresses the importance of distinguishing grief from depression. In addition, some authors suggest that depression after bereavement is qualitatively and prognostically different from depression at other points in the life course which raises uncertainty over the need for treatment [23].

A central argument for removing the depression exclusion in DSM-5 was that bereavement related depression is not meaningfully different from other major depressive episodes and hence exclusion has no scientific or clinical basis [4], [24]. The similarity of most predictors of prescribing in bereaved and non-bereaved individuals, in particular previous history of depression and comorbidity, is reassuring. However, the marked difference in the effect of age raises some concern that prescribing may indicate treatment of grief rather than depression. It could be argued that depression is known to be under-diagnosed in older people and the increased recognition after bereavement may be helpful in addressing this concern. However, the potential for misclassification of patients with grief and depression may mean such increased recognition is counter-productive and not necessarily benefical.

Although our study cannot comment directly on use of non-drug psychological therapies, there may be a role for such therapy in line with stepped care guidance for depression, in bereaved individuals in place of antidepressant therapy or as preventive therapy [25].

The common use of psychotropic medication after bereavement raises concerns over social and cultural iatrogenesis with medication viewed by patients, clinicians, families and society as an easy, and potentially cheaper, substitute for social or psychological support and professional time [26]. Society and health services need to consider whether current medication initiation rates are desirable and whether there are better ways to respond to the needs and distress of bereaved individuals.

## Supporting Information

Table S1 **Adjusted Hazard Ratios for Predictors of Initiation of Hypnotics & Anxiolytics (within 2 months) and Antidepressants (within 6 months) in Matched Non-Bereaved Individuals (n = 59,280).**
(DOCX)
