# Supplementary material for: Initiation of Psychotropic Medication after Partner Bereavement: A Matched Cohort Study
Source: PLoS One. 2013 Nov 5;8(11):e77734. doi: 10.1371/journal.pone.0077734 (PMC3818377; doi:10.1371/journal.pone.0077734)
Supplement: Table S1 — Adjusted Hazard Ratios for Predictors of Initiation of Hypnotics & Anxiolytics (within 2 months) and Antidepressants (within 6 months) in Matched Non-Bereaved Individuals (n = 59,280). (DOCX) [file pone.0077734.s001.docx]

**Table S1: Adjusted Hazard Ratios for Predictors of Initiation of Hypnotics & Anxiolytics (within 2 months) and Antidepressants (within 6 months) in Matched Non-Bereaved Individuals (n=59,280)**

| Group | **Total** | **Hypnotics & Anxiolytics** | | | | **Antidepressants** | | | |
| --- | --- | --- | --- | --- | --- | --- | --- | --- | --- |
|  |  | Initiators | % | **HR^a^** | 95%CI | Initiators | % | **HR^a^** | 95%CI |
|  |  |  |  |  |  |  |  |  |  |
| Gender |  |  |  |  |  |  |  |  |  |
| Men | 23,815 | 113 | 0.5% | **1** | _ | 190 | 0.8% | **1** | _ |
| Women | 35,465 | 216 | 0.6% | **1**.**41** | 1.12 to 1.78 | 413 | 1.2% | **1**.**62** | 1.36 to 1.94 |
|  |  |  |  |  |  |  |  |  |  |
| Age at Bereavement |  |  |  |  |  |  |  |  |  |
| 60-64 | 4,135 | 25 | 0.6% | **1.08** | 0.69 to 1.69 | 30 | 0.7% | **0.73** | 0.49 to 1.08 |
| 65-69 | 9,922 | 50 | 0.5% | **0.87** | 0.61 to 1.23 | 79 | 0.8% | **0.77** | 0.59 to 1.02 |
| 70-74 | 13,317 | 54 | 0.4% | **0.68** | 0.48 to 0.95 | 108 | 0.8% | **0.77** | 0.60 to 0.99 |
| 75-79 | 14,389 | 88 | 0.6% | **1** | _ | 151 | 1.0% | **1** | _ |
| 80-84 | 11,418 | 67 | 0.6% | **0.93** | 0.67 to 1.28 | 144 | 1.3% | **1.25** | 0.99 to 1.57 |
| 85-89 | 6,011 | 45 | 0.8% | **1.24** | 0.86 to 1.79 | 91 | 1.5% | **1.59** | 1.22 to 2.08 |
|  |  |  |  |  |  |  |  |  |  |
| Townsend Qunitile |  |  |  |  |  |  |  |  |  |
| 1 (Least Deprived) | 18,749 | 97 | 0.5% | **1** | _ | 148 | 0.8% | **1** | _ |
| 2 | 15,724 | 88 | 0.6% | **1**.**10** | 0.82 to 1.46 | 169 | 1.1% | **1**.**32** | 1.06 to 1.64 |
| 3 | 11,524 | 61 | 0.5% | **1**.**02** | 0.74 to 1.41 | 142 | 1.2% | **1**.**50** | 1.19 to 1.89 |
| 4 | 8,223 | 52 | 0.6% | **1**.**19** | 0.84 to 1.67 | 73 | 0.9% | **1**.**03** | 0.78 to 1.37 |
| 5 (Most Deprived) | 3,872 | 26 | 0.7% | **1**.**27** | 0.82 to 1.97 | 64 | 1.7% | **1**.**86** | 1.38 to 2.52 |
| missing | 1,188 | 5 | 0.4% | **0**.**85** | 0.34 to 2.10 | 7 | 0.6% | **0**.**74** | 0.34 to 1.57 |
|  |  |  |  |  |  |  |  |  |  |
| Charlson Index |  |  |  |  |  |  |  |  |  |
| 0 | 29,703 | 138 | 0.5% | **1** | _ | 244 | 0.8% | **1** | _ |
| 1 | 14,000 | 82 | 0.6% | **1**.**26** | 0.96 to 1.66 | 146 | 1.0% | **1**.**22** | 0.99 to 1.50 |
| 2-3 | 12,799 | 76 | 0.6% | **1**.**27** | 0.95 to 1.69 | 162 | 1.3% | **1**.**46** | 1.19 to 1.78 |
| 4+ | 2,778 | 33 | 1.2% | **2**.**59** | 1.75 to 3.84 | 51 | 1.8% | **2**.**04** | 1.49 to 2.78 |
|  |  |  |  |  |  |  |  |  |  |
| Depression |  |  |  |  |  |  |  |  |  |
| No History | 54,490 | 289 | 0.5% | **1** | _ | 510 | 0.9% | **1** | _ |
| History | 4,790 | 40 | 0.8% | **1**.**52** | 1.09 to 2.12 | 93 | 1.9% | **2.04** | 1.63 to 2.55 |
|  |  |  |  |  |  |  |  |  |  |
| Bereavement Season |  |  |  |  |  |  |  |  |  |
| Non-Winter | 38,422 | 215 | 0.6% | **1** | _ | 378 | 1.0% | **1** | _ |
| Winter | 20,858 | 114 | 0.6% | **0**.**99** | 0.79 to 1.25 | 225 | 1.1% | **1**.**02** | 0.86 to 1.20 |
|  |  |  |  |  |  |  |  |  |  |
| Practice Prescribing^b^ |  |  |  |  |  |  |  |  |  |
| I | 11,295 | 47 | 0.4% | **1** | _ | 80 | 0.8% | **1** | _ |
| II | 12,902 | 64 | 0.5% | **1**.**10** | 0.75 to 1.61 | 126 | 0.9% | **1**.**12** | 0.84 to 1.48 |
| III | 12,215 | 59 | 0.5% | **1**.**10** | 0.74 to 1.63 | 150 | 1.2% | **1**.**49** | 1.13 to 1.96 |
| IV | 13,650 | 78 | 0.6% | **1**.**35** | 0.93 to 1.95 | 138 | 1.1% | **1**.**41** | 1.06 to 1.87 |
| V | 9,218 | 81 | 0.9% | **1**.**89** | 1.28 to 2.80 | 109 | 1.2% | **1**.**52** | 1.11 to 2.08 |
|  |  |  |  |  |  |  |  |  |  |

^a^Hazard ratios adjusted for all determinants in table, UK Region and Year

^b^ Quintiles of annual practice prescribing to non-bereaved couples (% of patients in receipt of this drug class)
